# A Case of Severe and Refractory Membranous Nephropathy Associated to Mucous Membrane Pemphigoid

**DOI:** 10.1155/2021/9940293

**Published:** 2021-05-11

**Authors:** Eric Mereniuk, Laura Sabbah, Jean-Paul Makhzoum

**Affiliations:** ^1^Department of Medicine, Université de Montreal, Montreal, Canada; ^2^Division of Dermatology, Department of Medicine, Hôpital du Sacré-Coeur de Montréal, Université de Montreal, Montreal, Canada; ^3^Vasculitis Clinic, Department of Medicine, Hôpital du Sacré-Coeur de Montréal, Université de Montreal, Montreal, Canada

## Abstract

Primary membranous nephropathy (MN) and mucous membrane pemphigoid (MMP) are two autoimmune conditions with well-defined diagnostic and treatment guidelines. MN has been linked to bullous pemphigoid (BP) in certain case reports, though little is known regarding the association of MN and other bullous diseases. The association of MN and MMP has rarely been described, and very little data exist regarding the treatment of this association. We report a case of severe refractory membranous nephropathy secondary to mucous membrane pemphigoid successfully treated with rituximab. A 35-year-old woman with known MMP was referred to our clinic for new-onset generalized edema and proteinuria. MN was confirmed on renal biopsy. Despite therapy with high-dose systemic glucocorticoids, combined with mycophenolate mofetil, and later azathioprine, nephrotic-range proteinuria persisted even at a daily dose of prednisone of 40 mg. The patient was then started on rituximab infusions, which induced remission of both mucous membrane pemphigoid and membranous glomerulonephritis. This suggests that MN can be secondary to MMP, and rituximab may be useful induce remission in cases that are refractory to standard therapy.

## 1. Introduction

Membranous nephropathy (MN) is the most common cause of nephrotic syndrome in adults and is most commonly primary. MN may be associated with hepatitis B, autoimmune diseases, malignancy, and certain drugs [1, 2]. Bullous pemphigoid (BP) is an acquired autoimmune bullous disease characterized by tense blisters with serous or hemorrhagic content arising on the skin or on mucous membranes, whereas mucous membrane pemphigoid (MMP) involves predominantly mucous membranes, most commonly the oral cavity and conjunctivae [3, 4]. MN associated with BP has been reported. However, only one case of concomitant MN and MMP has been described [5]. The appropriate management of MN associated with MMP is therefore unknown. We report the case of a patient with a severe refractory case of MN associated with MMP who was successfully treated with rituximab.

## 2. Case Presentation

A 35-year-old Caucasian woman was referred to our clinic for refractory generalized edema and nephrotic syndrome of unclear etiology. Prior medical history was significant for MMP and a benign ovarian cyst. Family history was significant for renal cancer and ankylosing spondylitis.

**Figure 1:**
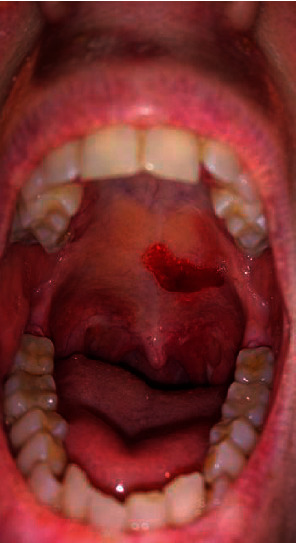
Severe erosions and presence of bullous lesions of the oral mucosa.

The patient had initially been referred to dermatology after developing bullous lesions and erosions of her oral mucosa and to a lesser degree of her skin (Figure 1). Biopsy revealed a subepidermal blister and linear deposits of IgG and C3 along the basement membrane on direct immunofluorescence. The patient was also found to have circulating antibasement membrane antibodies, confirming the suspected diagnosis of MMP. Topical glucocorticoid treatment was initiated, with little clinical response. High-dose systemic glucocorticoids were then started to induce disease remission.

Three months later, the patient had presented new-onset generalized edema and foamy urine. Initial workup showed nephrotic-range proteinuria (18 grams per 24 hours) with normal creatinine clearance. The patient was promptly referred to a nephrologist for investigations and renal biopsy. Renal biopsy revealed MN with immunofluorescence showing IgG-type antibodies and C3 deposits in a pseudolinear pattern along glomerular capillary membranes with immune complex deposits. PLA2R expression was absent on kidney biopsy.

Initial assessment was remarkable for anasarca and mild livedo reticularis on the upper and lower limbs. There was no history of symptoms suggestive of underlying connective tissue disease or systemic vasculitis. Routine laboratory workup showed a severe hypoalbuminaemia (20 g/L) but was otherwise unremarkable, including negative HIV, hepatitis B, and hepatitis C. Serological testing for connective tissue disease and vasculitis was unremarkable with negative antinuclear antibodies (ANA), extractable nuclear antigens (ENA), dual-stranded DNA antibody (anti-DNA), rheumatoid factor (RF), antineutrophil cytoplasmic antibodies (ANCA), glomerular basement membrane antibody (anti-GBM), C3-C4, myositis panel, antiphospholipid panel, and cryoglobulins. Serum protein electrophoresis and serum light chains did not reveal a monoclonal gammopathy. Serum anti-PLA2R antibodies were found to be negative. Computerized tomography (CT) scan of the thorax and abdomen was unremarkable and did not show any sign of malignancy. A diagnosis of MN secondary to MMP was made.

**Figure 2:**
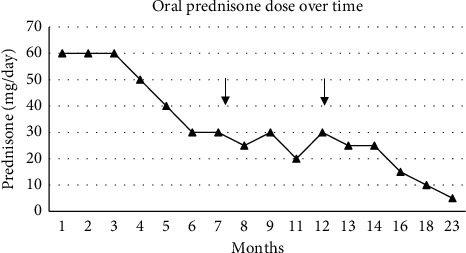
Oral prednisone dose over time. The arrow represents initiation of rituximab.

**Figure 3:**
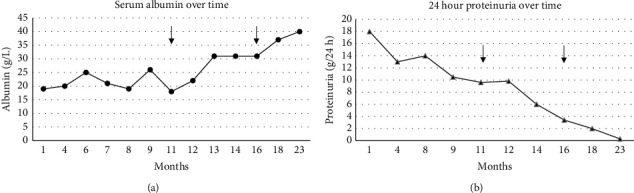
Serum albumin over time (top panel) and 24-hour proteinuria over time. The arrow represents infusions of rituximab.

After a two-month course of high-dose oral prednisone (1 mg/kg), edema was markedly reduced, and serum albumin had increased. Mycophenolic acid (MPA) was initiated to address both MN and MMP and titrated to reach 1080 mg twice daily, with a progressive tapering of oral glucocorticoids (Figure 2). Initial laboratory follow-up showed reduced proteinuria (Figure 3).

Six months later, while on oral prednisone (20 mg/day) and MPA (2160 mg/day), the patient presented a relapse of MMP and anasarca. Oral glucocorticoids were increased, MPA was stopped, and the patient was started on azathioprine which was rapidly stopped due to gastrointestinal side effects.

Rituximab infusions of 375 mg/m^2^ weekly for four doses were prescribed due to lack of response to mycophenolate mofetil of both MMP and MN and intolerance to azathioprine. On assessment 4 months after starting rituximab, clinical remission was observed, with absence of edema, resolution of cutaneous symptoms, and reduction of proteinuria (Figure 3). A second cycle of rituximab (375 mg/m^2^ weekly for four weeks) was administered 6 months following the first to maintain cutaneous remission and further induce MN remission. On subsequent follow-up 12 months after starting rituximab, cutaneous remission was maintained, proteinuria had completely resolved, and the patient's serum albumin was normal, which allowed complete discontinuation of oral prednisone. Although B-cell repopulation was present 12 months following the last rituximab infusion, the patient remains in complete remission 18 months after her last infusion.

## 3. Discussion

MN is most often primary and has been linked to phospholipase A2 receptor antibodies (PLA2R) in up to 80% of patients with this condition [1]. Therapy for severe MN includes high-dose glucocorticoids and cyclophosphamide. However, recent evidence suggests that the monoclonal anti-CD20 antibody rituximab is equivalent to cyclophosphamide for the remission induction and may reduce proteinuria relapse during extended follow-up [4, 6].

MN associated with BP has previously been reported. Histological similarities exist between MN and BP, with linear IgG and C3 deposits playing a key role in both conditions [1, 4]. The exact mechanism of MN associated with BP is not precisely known. However, the pathogenesis of BP involves autoantibodies (mainly IgG, though certain forms of IgA- or IgE-related disease have been documented) directed at epidermal basement membrane proteins BP180 (collagen XVII) and BP230 (BPAG1). Recent data have shown BP180 expression in the kidney as well and could explain the overlap in these two diseases [4].

Our patient presented with MN associated with MMP, in which mucosal lesions are predominant as opposed to BP [3]. MMP, in addition to expression of antibodies against BP180 and BP230, is also driven by the expression of antibodies targeting laminin-332, collagen VII, and *α*6*β*4 integrin. These proteins, expressed in deeper layers of the epithelium, have been proposed mechanisms behind MMP's predominant mucosal lesions, as well as its increased incidence of scarring lesions when compared to BP [5]. As with BP, the expression of antibodies against BP180 could explain the development of renal disease in MMP. Furthermore, *α*6*β*4 integrin has been found to be expressed in renal cells and could contribute to MN in patients with MMP [7].

One case of concomitant MN and MMP has been reported, and very little is known about this association and its optimal management [5]. Certain similarities between this case and our patient exist, most notably the presence of linear IgG and C3 deposits on both skin and kidney biopsies and an extensive workup revealing no alternative explanation to the patient's MN (including negative PLA2R antibodies). Furthermore, both cutaneous and renal flares coincided, as was the case in our patient. However, our patient differs from this case in the severity of renal disease and the presence of nephrotic-range proteinuria.

Mild MMP is usually managed with topical glucocorticoids, while severe cases are typically treated with high-dose systemic glucocorticoids, followed by other systemic medications such as mycophenolate mofetil or azathioprine to maintain disease remission.

The optimal treatment strategy for MN associated with BP is unknown, though conventional therapies combine glucocorticoids to achieve rapid remission, followed by either mycophenolate mofetil or azathioprine as a glucocorticoid-sparing agent [4]. Our patient did not respond to conventional therapy and was therefore started on a course of rituximab, which had been reported as an effective treatment MN associated with BP [5, 6, 8–10].

Rituximab, a monoclonal anti-CD20 antibody, was able to induce complete remission of both MMP and MN in our patient. B-cell abnormalities and the resulting production of antibodies have been suggested to contribute to the pathogenesis of MN, BP, and MMP [6, 8, 10]. This offers an explanation for rituximab's efficacy in treating numerous autoimmune diseases, particularly in their IgG-dependent forms, as the pathogenesis of these diseases may further depend on B-cell abnormalities.

## 4. Conclusion

MN can be secondary to MMP, and rituximab may be useful induce remission in cases that are refractory to standard therapy. Long-term follow-up is necessary to further assess prognosis, risk of relapse, and the need for subsequent maintenance infusions of rituximab.

## Data Availability

The datasets used and/or analyzed during the current study are available from the corresponding author on reasonable request.
